# Coral population trajectories, increased disturbance and management intervention: a sensitivity analysis

**DOI:** 10.1002/ece3.519

**Published:** 2013-03-07

**Authors:** Bernhard Riegl, Michael Berumen, Andrew Bruckner

**Affiliations:** 1 National Coral Reef Institute, Nova Southeastern UniversityDania, Florida, USA; 2 Red Sea Research Center, King Abdullah University of Science and TechnologyThuwal, KSA; 3 Khaled bin Sultan Living Oceans FoundationAndover, MD, USA

**Keywords:** Coral population dynamics, coral reef, global change, impacts, management, predator outbreak, sensitivity

## Abstract

Coral reefs distant from human population were sampled in the Red Sea and one-third showed degradation by predator outbreaks (crown-of-thorns-starfish = COTS observed in all regions in all years) or bleaching (1998, 2010). Models were built to assess future trajectories. They assumed variable coral types (slow/fast growing), disturbance frequencies (5,10,20 years), mortality (equal or not), and connectivity (un/connected to un/disturbed community). Known disturbances were used to parameterize models. Present and future disturbances were estimated from remote-sensing chlorophyll and temperature data. Simulations and sensitivity analysis suggest community resilience at >20-year disturbance frequency, but degradation at higher frequency. Trajectories move from fast-grower to slow-grower dominance at intermediate disturbance frequency, then again to fast-grower dominance. A similar succession was observed in the field: *Acropora* to *Porites* to *Stylophora*/*Pocillopora* dominance on shallow reefs, and a transition from large poritids to small faviids on deep reefs. *Synthesis and application*: Even distant reefs are impacted by global changes. COTS impacts and bleaching were key driver of coral degradation, coral population decline could be reduced if these outbreaks and bleaching susceptibility were managed by maintaining water quality and by other interventions. Just leaving reefs alone, seems no longer a satisfactory option.

## Introduction

Coral reefs worldwide are impacted by global change - a combined multitude of large-scale and local stressors. Large-scale stressors are mainly related to climate change and find their expression in region-wide coral bleaching events (Baker et al. 2008) or decreased growth rate (Cantin et al. 2010). Local stressors, like localized pollution phenomena, can either be independent of large-scale issues or can be indirectly driven by them (Riegl et al. 2009). Sometimes, a connection exists. Outbreaks of the predatory crown-of-thorns starfish (*Acanthaster planci*, COTS) react sensitively to increased nutrient-levels that can be due to run-off, a local phenomenon, or excursions of oceanic mixing zones, a large-scale phenomenon (Birkeland 1982; Houk et al. 2007; Fabricius et al. 2010). Bleaching, whereby unusual heat causes corals to lose their symbiotic algae and die (Baker et al. 2008), is strongly affected by climate change. Whether management actions can or should be taken to combat these threats and whether it could alleviate reef degradation is a matter of debate (De'ath et al. 2012).

During disturbances, taxa vary in susceptibility and so do population dynamic responses. Winners and losers based on bleaching sensitivity (Marshal and Baird 2000; Loya et al. 2001) and predator preferences (Pratchett et al. 2009; Kayal et al. 2012) are known and resultant community changes have been postulated (Done 1999; Hoegh-Guldberg et al. 2007; Riegl and Purkis 2009). Done (1999) predicted and De'ath et al. (2012) demonstrated changes at reef to regional scales, determined by frequency and severity of disturbances. Many fast-growing and weedy species exist that specialize in rapidly exploiting free space (like *Acropora*, *Pocillopora*, etc.) but some of them have low persistence during disturbances (Darling et al. 2012). Massive species (notably *Porites*) are slow-growing and tend to survive better during bleaching or predation (Marshal and Baird 2000; Loya et al. 2001). This may afford them resistance to increased disturbance (Riegl and Purkis 2009), but may weaken their resilience if disturbances are very frequent, since they may not have enough time to regrow (Done 1999; Darling et al. 2012).

Such hypotheses can be field-tested in the Red Sea. Fast-growing species (*Acropora*, *Pocillopora*) dominate windward communities that differ from leeward and deeper communities of slower-growing species (*Porites*, faviids, and others; Schuhmacher and Mergner 1985; Sheppard and Sheppard 1991; DeVantier et al. 2000). Turn-over rates and successions are known (Reinicke et al. 2003). While the Red Sea is increasingly impacted by pollution, overfishing and a rapidly expanding human footprint, many offshore reefs have only sporadic direct human interference (Rowlands et al. 2012). However, even they are impacted by COTS outbreaks and bleaching. We characterized coral communities with transects, evaluated patterns of recruitment, counted disturbance incidence, and then used a mathematical model tuned to field measurements and disturbance predictions to examine the dynamics of the dominant coral types and whether management intervention would be effective to help avoid degradation.

## Material and Methods

**Figure 1:**
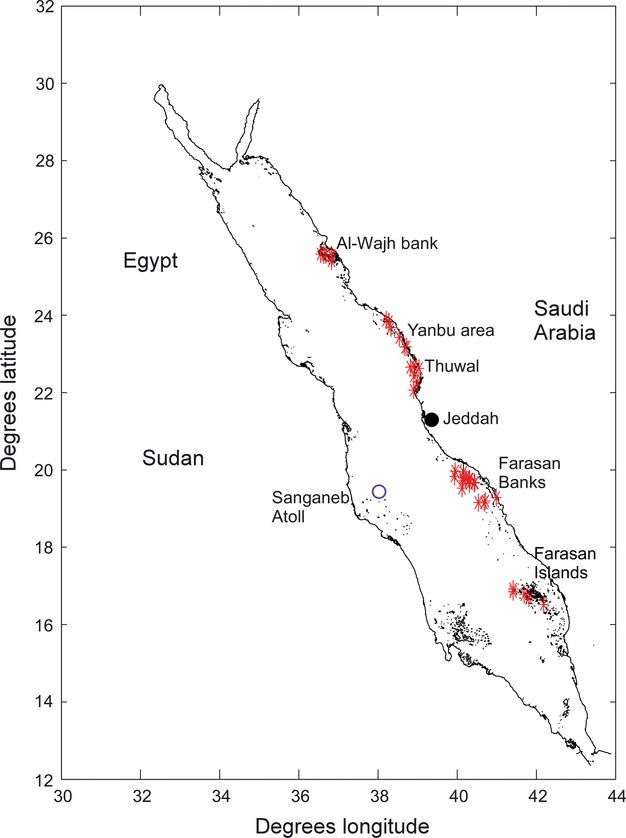
Sampling sites in the Red Sea. Red stars = transect sites for this study; blue circle = comparative site from the literature.

Reefs were sampled in Saudi Arabia between the Farasan Islands and Al Wajh Banks, by line-intercept and phototransects (Fig. 1). Sites were offshore, remote from human activities (habitation, fishing, etc.). Transects were placed in a stratified random pattern at depths centered on <5 m, 10 m, 20 m, 25 m. Overlapping photographs created 0.5 × 10 m photo-corridors. Images were merged and gridded to unit pixel-size (1 pixel = 1 mm^2^). Intercepts of all corals along a center-line were measured to the nearest cm. 163 transects were evaluated. In 2010, bleaching was assessed with 10 m line transects at 5, 10, 15 m.

### Transect evaluation

Agglomerative, hierarchical cluster-analysis (Ward's method of linkage, Euclidian distance) was used for grouping transects. A more detailed analysis of community pattern is published elsewhere (Riegl et al. 2012). The cophenetic correlation index was used to optimize cluster performance (Jongman et al. 1995). To test how disturbances acted across regions, spatial autocorrelation in coral cover among sites was calculated. Only region-wide disturbances were expected to give strong spatial autocorrelation of cover. Structure and correlation in variograms were evaluated (Jongman et al. 1995). Absence of spatial autocorrelation was considered evidence of primarily local factors determining cover.

### Recruitment counts

In 2009, all corals <5 cm diameter were counted within 25 × 25 cm squares (*N* = 580), five of which were placed haphazardly within a phototransect. Counts of small juvenile corals and recruits were aided by coral fluorescence, which enhanced detection under a UV light (Baird et al. 2006). These counts were also used to calculate recruitment rates (see below).

### Modeling approach

We modeled two coral types in three life-stages (Sebens 1982; Riegl and Purkis 2009). All variables of interest (disease and predation mortality, growth into next size class, immigration/emigration of spat) were made explicit to allow manipulation. Within the discrete stages, a continuous time model was used to express cover changing continuously over time. Fast-growing (*Acropora, Pocillopora*) and slow-growing corals (*Porites*, faviids) were considered in three stages (fast: small = recruit *C*_1_, medium *C*_2_, large *C*_3_; slow: *C*_4_, *C*_5_, *C*_6_) which allowed assigning functional differences like fertility level, competitiveness, etc., to each stage while allowing continuous growth within each stage and graduation among stages (determined by *G*_*i*_). An aggressive hierarchy in direct encounters was assumed, with fast growers winning (Lang and Chornesky 1990). Many Red Sea corals have extended recruitment periods, making the continuous model appropriate for generation of the smallest size-class. Medium and large colonies were considered to reproduce sexually and asexually, with exponentially higher fecundity in large colonies (Hall and Hughes 1996). Sexual recruits differed from asexual in their ability to migrate between populations. Coral populations were considered either self-seeding and/or to receive larvae from and donate to connected populations (via self-seeding parameter *ss*). Recruits/small corals were assumed to compete among types but not able to displace established larger corals (Lang and Chornesky 1990; Caley et al. 1996) resulting in a fixed and uniform carrying capacity (*K*) for all species. External, pulsed mortality due to disturbances was introduced as multiplication factor <1 after a certain number of time-steps (years between disturbance).

Fast-growers:


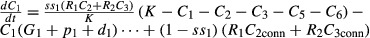
(1)


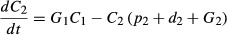
(2)


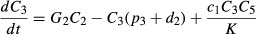
(3)

Slow growers:


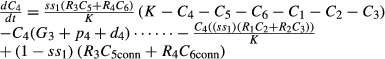
(4)


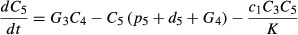
(5)


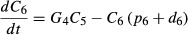
(6)

Coral-type specificity of parameters *R*, *K*, *d*, *G*, *c*, *p,* and *ss* is indicated by subscripts. *C*_*j*_ is coral cover in stage *j*, *K* is carrying capacity (*C*_*i*_ fitting in system without causing negative growth-rates), *G*_*j*_ is growth (small colonies growing into large), *d*_*i*_ and *p*_i_ are mortality parameters caused by disease or non-outbreak predation, *c* is a competition parameter, *ss* is the self-seeding parameter, therefore 1-*ss* is an emigration value scaling transfers to and from connected outside populations (*C*_*j*conn_).

The small stage is not yet fertile and competes for space among types (fast-growers win over slow-growers). Their rate of change in cover (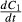) is a function of fertility of the bigger size classes (*R*_*i*_*C*_*j*_, *i* = 1,2,3,4, *j* = 2,3,5,6), where *R*_*i*_ is further scaled by a self-seeding term (*ss*), ranging 0–1, determining the ratio of recruits retained on the homreef (*ss*_*i*_*R*_*i*_*C*_*j*_). Import of larvae from a connected reef with similar dyanmics is possible ([1−*ss*_*i*_]*R*_*i*_*C*_*j*conn_, *j*conn referring to medium and large size-classes of connected population). Gamete survivability, post-settlement fate, as well as shrinkage from larger stages is included in *R*. Settlement is into free space (after subtraction of medium and large corals from *K*) but small fast-growers can overgrow slow-growers in the competition term *C*_4_*/K* (*ss*_1_[*R*_1_*C*_2_*+R*_2_*C*_3_]), where in brackets is the rate of production of small fast-growers (from eq. 1) that can interact with *C*_4_, the small slow-growers. This is the amount of space occupied by small slow-growers that are hit by a fast-grower recruit in that specific time-period. Multiple hits are unlikely in the short period *dt* (Sebens 1982). Losses occur by growth into the next-larger size class (*G*_*i*,_ *i* = 1,3), predation (*p*_*i*,_ *i* = 1,4) and diseases (*d*_*i*,_ *i* = 1,4).

The medium-sized stage is fertile (terms *R*_*i*_*C*_*j*_ in eqs. 1,4) and slow-growers lose in competition with large fast-growers (*c*_1_*C*_3_*C*_5_*/K* is subtracted from *C*_5_ and added to *C*_3_*,c* weakens the competitive effect). The competition term differs from that for small corals because delivery of medium and large corals is by growth not reproduction (*G*_*i*_ not *ss*_*i*_*R*_*i*_*C*_*j*_). Growth into the next size-class occurs continuously (*G*_*i*_). In the large size class, corals have reached a size refuge where they can no longer be displaced. Medium and large corals suffer losses by predators and diseases (*p*_*i*_ and *d*_*i*_, where *i* = 2,3 for fast-growers, 5,6 for slow-growers). Large corals are more fecund than the medium-sized stages (we derive fecundities from field data, see below). The upper population boundary (carrying capacity *K*) cannot be exceeded since it limits recruitment. But as a result of *K* only appearing in equation 1 and 4, large individuals tend to “stack up” with time, if disease and predation mortality (*d* and *p*) are low (van Sickle 1977) – and as is observed in real coral populations (Riegl et al. 2012).

It is well known (Hughes 1984) that their clonal structure allows corals to shrink to smaller “tissue size-classes” (amount of tissue remaining on a larger skeleton after shrinkage equivalent to that on a smaller skeleton) and even, by fragmentation, into smaller “skeleton size-classes.” This frequently happens in response to disturbances that cause mortality. We wrapped this shrinkage implicitly into the stage-specific mortality and survival at disturbances, smaller stages having higher survival rate than larger stages at disturbances, which includes cover received by shrinkage from larger stages besides small colonies bleaching less (Loya et al. 2001; Shenkar et al. 2005). If a connected population was assumed, equations 1–6 were simultaneously solved for the two (or more) locations with identical parameter values and connectivity via parameter *ss*. Numerical approximations were implemented by fourth-order Runge-Kutta methods.

### Sensitivity analysis and model parameterization

General sensitivity analysis of a similar model is presented in Riegl and Purkis (2009). Model outcomes are most sensitive to spacing and severity of disturbances and levels of recruitment. Hence, we employ a sensitivity analysis with regards to disturbance frequency and severity to project changes. Mortalities were introduced as scalar multipliers of the population variable. Three disturbance frequencies (5,10,20 years) were used. Scenarios of connected populations assumed that only the focal population experienced mortality (multiplication of population at disturbance by <1) but not the connected (multiplication by 1). Or both could be disturbed. Three mortality scenarios were assumed: mild, medium, and severe (Table 1).

**Table 1:** Parameter values used for model. Mortality introduced as a multiplier of coral cover at variable intervals. At disturbance (each 5, 10, 20 years), population multiplied by values under “Disturbance Scenario.” In equal-mortality-scenarios (lower bound of outcome cone, Fig. 9) both fast- and slow-growers were multiplied by values shown for fast-growers. Upper bound of outcome cones in Fig.9 calculated with values shown here. In connected scenarios assuming only local impacts, focal population multiplied by values below, connected population multiplied by 1

	Fast-growing corals	Slow-growing corals	Dimension (Unit)
Relative space cover at onset of model	7	1	Cover/area (m^2^)
*K*	250.000 (fast + slow)	250.000 (fast + slow)	Area (m^2^)
*R*_medium_	*R*_2_ = 0.5	*R*_5_ = 0.8	1/time
*R*_big_	*R*_3_ = 1	*R*_6_ = 1.25	1/time
*c* (competition)	0.02	0 or 0.02	1/time
*G* (growth among stages 1–2)	0.8	0.2	1/time
*G* (growth among stages 2–3)	0.4	0.1	1/time
*d* (background disease incidence)	0.015	0.01	1/time
*p* (background predation incidence – outside of outbreak)	0.01	0.01	1/time
*ss* (recruit retention on home reef)	0 (isolated)–0.5 (connected)	0 (isolated)–0.5 (connected)	Dimensionless multiplier of 1/time
Disturbance scenarios:			
Mild mortality			
Small corals	0.8*C*_1_	0.95*C*_4_	Dimensionless multiplier × cover/time
Medium corals	0.7*C*_2_	0.95*C*_5_	Dimensionless multiplier × cover/time
Large corals	0.7*C*_3_	0.95*C*_6_	Dimensionless multiplier × cover/time
Moderate mortality			
Small corals	0.6*C*_1_	0.85*C*_4_	Dimensionless multiplier × cover/time
Medium corals	0.5*C*_2_	0.75*C*_5_	Dimensionless multiplier × cover/time
Large corals	0.5*C*_3_	0.75*C*_6_	Dimensionless multiplier × cover/time
Severe mortality			
Small corals	0.4*C*_1_	0.5*C*_4_	Dimensionless multiplier × cover/time
Medium corals	0.3*C*_2_	0.4*C*_5_	Dimensionless multiplier × cover/time
Large corals	0.3*C*_3_	0.4*C*_6_	Dimensionless multiplier × cover/time

Models were groundtruthed against field observations (parameters in Table 1). A reef area of 500 × 500 dimensionless units was assumed. Each spatial unit was allowed to accommodate one coral. Simulations started with a uniform cover of 0.005% of *K* in all life stages and let models run to near equilibrium (*dC*_*j*_/*C*_j_dt = 0). Population/community characteristics at that point were equivalent to the median of cover values observed on reefs (comparison of theoretical cover in life-form groups with transect results obtained from the field, see Results). External perturbations via a multiplier <1 (defining mortality at each life stage) were introduced after variable undisturbed model periods. While *K* corresponded strictly to a theoretical reef, realistic coverage (*C*_*j*_) and recruitment values (*R*_*i*_) were obtained. *R*_*i*_ was estimated from recruitment data obtained on the Farasan Banks in 2009, as ratio of small to large colonies across all transects and corresponding recruit counts. Exponential increase in fertility among coral sizes was assumed. Geometric mean of *R* assigned to size-classes was equal to reproductive rate estimated from transects. Growth rates in fast-growers were assumed four-times that of slow growers (*Porites, Favia, Platygyra* ∼1–2 cm, *Pocillopora* ∼4 cm, *Acropora* ∼10 cm; Dullo 2005). It was assumed that a slow-grower will require ∼5 years until puberty (Shlesinger and Loya 1991), resulting in annual 20% (*G* = 0.2) of the population graduating into the next size class. Consequently, *G* = 0.8 for fast-growers. The continuous model solutions were interpolated to annual steps for ease of visualization of the modeling period.

### Estimation of survival/mortality rates

In 2010, reefs were examined off Thuwal during and after a bleaching event (Fig. 1) with line transects to quantify bleached coral and track recovery (see also Furby et al. 2013). In all other areas, whenever encountered (2006–2010) COTS were evaluated in density/100 m-square (i.e. 10.000 m^2^) and damage to corals was estimated as percentage of corals eaten. Mortality rates assumed in the models (*d*, *p* and bleaching mortality multiplier of *C*_*i*_) correspond to mortality rates from reefs in 2006–2010.

### FG-index and size-index

We defined a dominance-index as ratio of modeled cover in fast- over slow growers. FG = 1 suggests equal cover, FG > 1 fast-grower dominance. Changes were expressed as units of ratio of dominance and not simply by changes in cover since variability in coral cover exists among reef sites (Bruno and Selig 2007; Baker et al. 2008), low cover is not necessarily correlated with degraded reef systems (Vroom 2010) and significant asynchrony exists within and among reefs with regards to cover changes (Bruno and Selig 2007). Permanent changes in the ratio of constituent species are usually seen as sign of degradation (Rogers and Miller 2006). To also evaluate sensitivity of relative coral size to disturbances, the ratio of small to big corals ([*C*_1_*+C*_2_*+C*_4_*+C*_5_]/[*C*_3_*+C*_6_]) was plotted through time. Changes in size-distributions are considered important indicators of changing ecological function (Bak and Meesters 1999).

### Oceanographic and temperature data

Temperature and nutrient dynamics was examined using Pathfinder 5 sea surface temperature (SST) and Aqua-Modis chlorophyll data (http://coastwatch.pfeg.noaa.gov/erddap), daily atmospheric temperature data and monthly HadISST (Rayner et al. 2003) SST data (http://climexp.kmnl.nl, and http://badc.nerc.ac.uk, crown copyright). We plotted chlorophyll levels and calculated daily, monthly, seasonal, and annual temperature anomalies from station data. Summer anomaly uses the mean of monthly anomalies for July–September. Annual mean anomaly uses the mean of twelve monthly anomalies, referenced to seasonal means.

## Results

### Community differentiation

**Figure 2:**
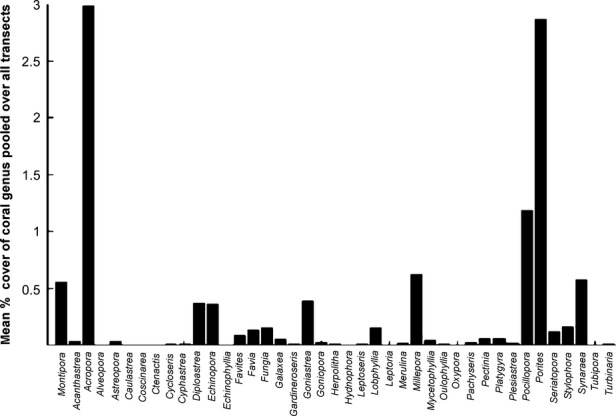
Mean cover over all transects in Saudi Arabia. The dominant genera are *Acropora*, *Porites,* and *Pocillopora*.

Most frequent genera were *Porites* and *Acropora*, with *P.lutea* the most frequent species (Table 2, Fig. 2). *Pocillopora* was also locally common and zone-forming at Yanbu and Wajh Bank. *Stylophora pistillata* was common and widespread in a variety of habitats, but zone-forming primarily in inshore environments and the Farasan Islands. In shallow transects, the three consistently dominant coral genera over all pooled sites were fast-growing *Acropora* (average cover over all transects 3%), slow-growing *Porites* (2.75%), and fast-growing *Pocillopora* (1.3%).

**Table 2:** Species dominance in Saudi Arabia. Relative contribution to coral cover in transects from the area

Farasan Islands	Farasan Banks	Yanbu area	Al Wajh Bank
*Porites lutea* (20%)	*Porites lutea* (19%)	*Pocillopora verrucosa* (27%)	*Porites lutea* (14%)
*Acropora clathrata* (14%)	*Porites rus* (9%)	*Porites lutea* (23%)	*Porites columnaris* (12%)
*Acropora forskali* (7%)	*Acropora pharaonis* (7%)	*Pocillopora damicornis* (6%)	*Acropora forskali* (10%)
*Montipora monasteriata* (7%)	*Acropora clathrata* (6%)	*Acropora gemmifera* (5%)	*Favia stelligera* (6%)
*Acropora cytherea* (4%)	*Diploastrea heliopora* (6%)	*Acropora lamarcki* (4%)	*Acropora lamarcki* (6%)

A cluster analysis of untransformed intercept data of genera (all species covers had been summed to give cover by genus) showed no difference among regions (no region formed unique clusters) but suggested a gross community differentiation into reefs dominated by *Acropora* and *Pocillopora* (windward) and/or *Porites* (leeward and deeper), as previously described (Schuhmacher and Mergner 1985; Sheppard and Sheppard 1991; Riegl et al. 2012). Thus, two functional types of dominant corals could be considered for models: fast- and slow-growing. Among the fast-growers, *Acropora* and *Pocillopora* showed different dynamics. *Pocillopora* dominated the most ephemerous and exposed habitats (reef flats and edges) together with a subset of *Acropora* (e.g. *A. humilis* group, *A. cytherea* group) and faviids. Large open-arborescent and corymbose *Acropora* dominated on reef slopes and lagoonal habitats. Large slow-growing poritids dominated in low-disturbance environments, such as fore-reef areas, reef slopes, and sheltered reef edges. These corals conform to life-history classifications (Darling et al. 2012) as “competitive” (*Acropora*), “weedy” (*Pocillopora*), grouped here as “fast-growing,” and “stress-tolerant” (*Porites*), here called “slow-growing.”

**Figure 3:**
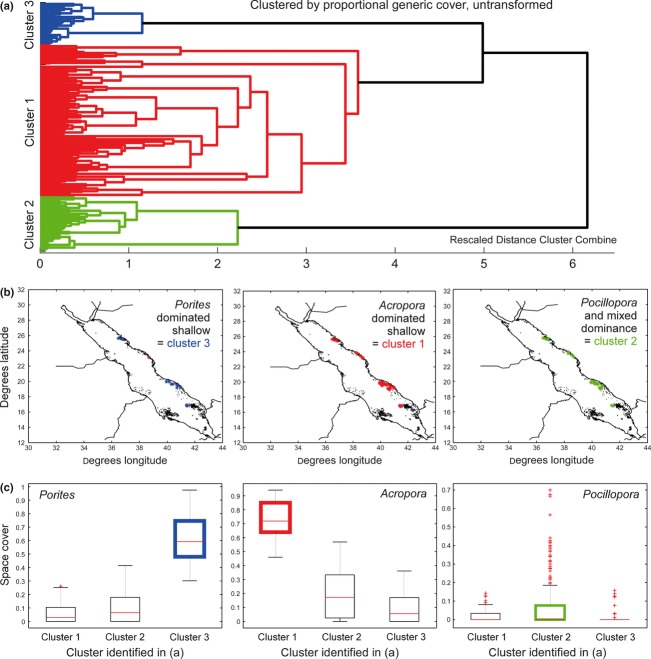
(a) Dendrogram of transect similarity, cluster levels optimized by maximizing cophenetic index (not shown). Euclidian distance and Ward's method of linkage. Three clusters correspond to coral communities dominated by fast-growers (*Acropora* and *Pocillopora*) or slow-growers (*Porites*). (b) geographic location of transects in clusters. No geographic separation of samples occurred, so community differentiation is similar across study area. (c) Box-and-whisper plots of relative cover by dominant coral types in each cluster (color-coded).

More transects fell within *Acropora* and *Pocillopora* dominance (Fig. 3) because sampling had concentrated on ocean-facing reefs with higher *Acropora* cover. Reef crests, shallow reef edges and recently disturbed reefs were dominated by small *Acropora* (mainly corymbose, clumping species) and *Pocillopora* or *Stylophora* (in the Farasan Islands, *Stylophora* took the place of *Pocillopora*, which was rarer in the S). Shallow windward communities showed *Acropora*-dominance in an intermediate-disturbance setting, consisting of large colonies (usually tabular or large, open arboresent stands) with strong admixture of slow-growing species (*Porites* spp., *Favia stelligera*, *Pavona maldivensis*). One cluster was made up of a mixed group of transects from *Pocillopora*-dominated sites and some semi-exposed *Millepora*-dominated sites (Fig. 3B). While it had greater mean depth than the *Acropora* and *Porites* dominated clusters, differences were not significant. *Porites* dominated transects were from leeward shallow or deeper sites (Fig. 3C).

### Recruitment

**Figure 4:**
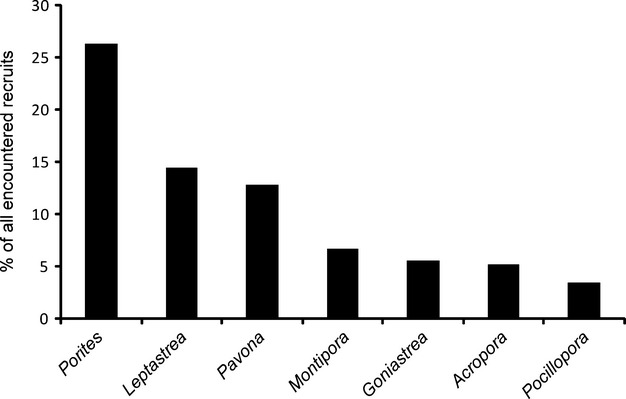
Seven genera made up ∼75% of all recruits encountered on the Farasan Banks. Data pooled across all depths.

On the Farasan Banks 3.3 ± 1.7 juveniles per 25 cm^2^ sampling unit suggested ∼13 juvenile corals (<5 cm diameter)/m^2^. Poritids were the most common recruiters (26%), followed by faviids (20%), *Pavona* (12%), and acroporids (11%). Seven genera made up three quarters of recruits (74.7%, Fig. 4). *Millepora* was encountered only once, making this the rarest recruiter of species commonly encountered as large corals. Distribution of recruitment reflected dominance of large corals: *Porites* recruited more commonly than *Acropora* and also covered more space on reefs. Commonly recruiting faviids, with comparable growth rates to *Porites*, covered less space due to their small size. Almost the entire encountered *Leptastrea* population consisted of small colonies. Although fast-growing *Montipora* recruited more frequently than *Acropora*, it covered less space which suggests higher mortality. Relative recruitment rate (*R*_*i*_ of eqs. 1 and 4) calculated as ratio of recruits in squares to big colonies in associated phototransecs was 0.64 for *Acropora,* 0.68 for *Pocillopora*, 1.12 for *Goniastrea,* and 0.83 for *Porites*. No recruits at all were found for many, especially the rare, species. This may be due to heterogeneity of recruitment patterns in time and space and underlines difficulties of recruitment estimation in open marine populations.

### Disturbances

Damage due to bleaching or COTS existed in 36% of all sites, increasing toward S (Table 3). During 2006–2009, no bleaching was observed. In 1998 bleaching was recorded north of Yanbu (DeVantier et al. 2000) and in 2010 in the Thuwal/Yanbu/Wajh area. COTS outbreaks existed in 2006 in the Farasan Islands, in 2008 on Wajh banks, and in 2009 on Farasan banks. COTS counts ranged from 10 to 498/100 m-square. COTS damage to corals ranged from near-total devastation (cover per transect 0–2%) at the end of or immediately after an outbreak to 10–30% of all corals eaten during earlier outbreak phases. In earlier stages, or low-density infestations, mortality in *Acropora* exceeded that of *Porites* about threefold (see also Pratchett et al. 2009; Kayal et al. 2012). In high-density/late-stage infestations all corals were eaten (except *Diploastrea heliopora*).

**Table 3:** Impacts per study region. Note: last two columns not necessarily have same sum as previous three columns, since also sites without major COTS/bleaching damage were included

Study area	Number of sites	Sites with acute mortality due to COTS(%)	Sites with acute mortality due to bleaching(%)	Sites with severe mortality in the near past (likely COTS) (%)	Sites with population reduction predominantly in *Acropora* and fast-growers(%)	Sites with population reduction predominantly in *Porites* and slow-growers(%)
Wajh Bank - 2008	30	10	0	15	15	10
Thuwal-Yanbu area - 2010	8	0	100	0	50	50
Farasan Banks - 2009	53	10	0	24	32	9
Farasan Islands - 2006	10	0	0	50	25	25

**Figure 5:**
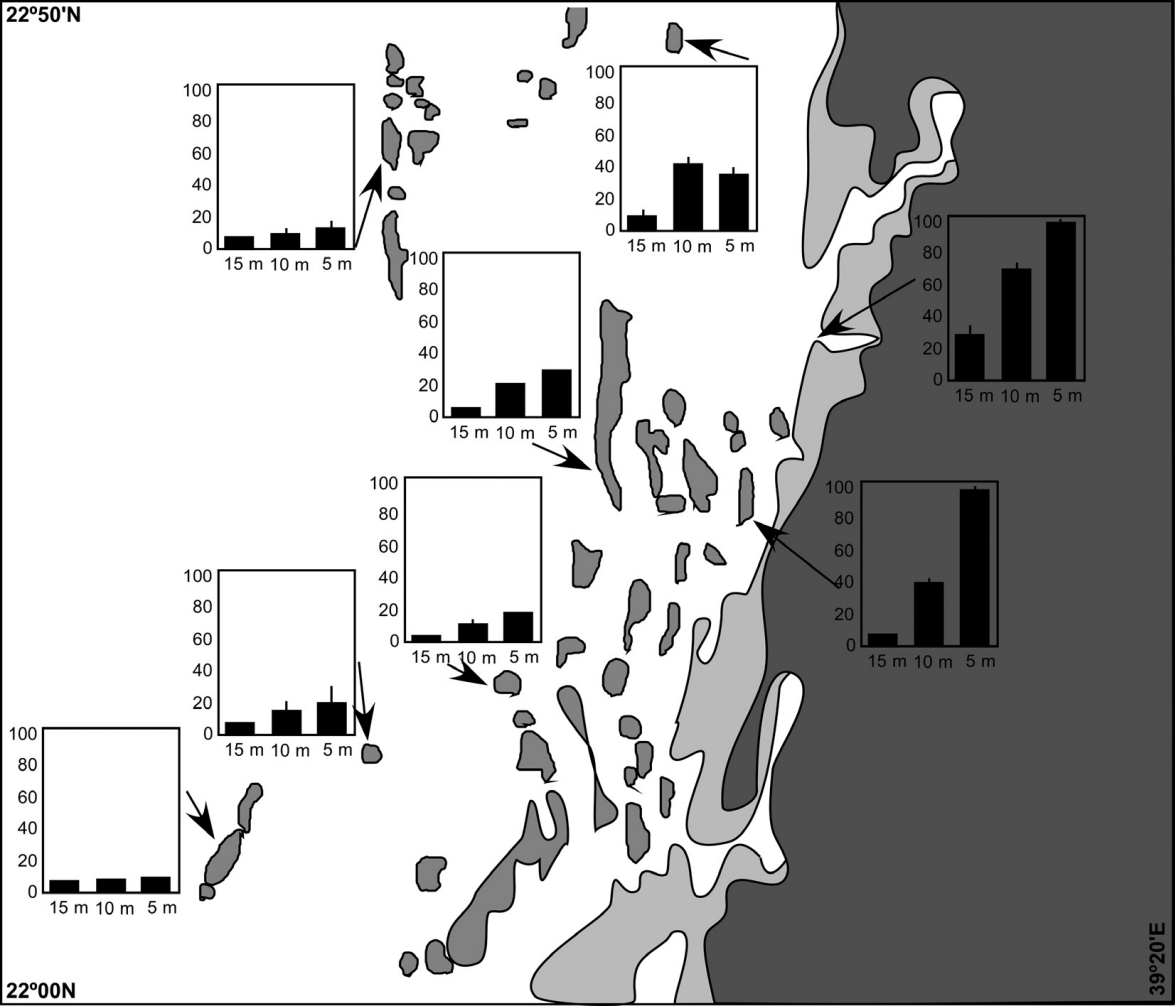
Relative bleaching incidence (y-axes) at Thuwal in 2010. Mortality in Table 4.

The 2010 bleaching affected offshore reefs less and mortality was lower than inshore (Fig. 5, Furby et al. 2013). Pattern of mortality varied among sites between fast- and slow-growing taxa (*Acropora*, *Pocillopora* vs. faviidae, *Porites*). On offshore reefs and in shallow water, mortality was in most instances (2/3 of cases) higher in the slow-growers, while on inshore reefs the situation tended to be the opposite (Table 4).

**Table 4:** Mortality pattern at Thuwal after the 2010 bleaching (see also Fig. 5) Unit = % mortality of corals

Depth(m)	Fast-growers Acroporidae	Fast-growers Pocilloporidae	Slow-growers Faviidae	Slow-growers Poritidae
Offshore 5	12.5	1.7	56.6	22.2
Offshore 10	15.6	1.0	17.9	15.5
Offshore 15	6.3	0.3	0	13.2
Inshore 5	100	95.5	99.4	85.1
Inshore 10	87	61.7	98	55.7
Inshore 15	22.7	19.7	20.5	40.8

Diseases occurred at low frequency (1% of slow-growers affected with *P. lobata* the most frequent, 1.5% fast-growers with *Acropora* the most frequent). White band, brown band, black band, skeleton-eroding black band were observed. Growth anomalies (“neoplasias”) existed sporadically.

Across all regions and years, ∼30% of reefs showed stress and mortality that could be attributed to predators and bleaching (Table 3). More sites showed reductions in fast-growers than slow-growers.

### Spatial pattern of disturbance

**Figure 6:**
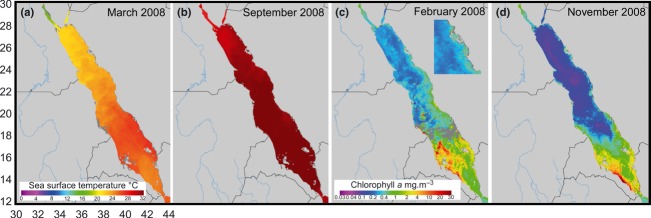
A) winter minimum B) summer maximum sea surface temperature in the Red Sea. C,D) Chlorophyll levels 4–6 months prior to surveys with COTS outbreaks. It takes COTS about this time to switch to corals as diet (Yamaguchi 1974; Houk et al. 2007). Inset in (C) shows elevated chlorophyll levels in Yanbu-Wajh region, 5 months prior to observed COTS infestations. Datasource: coastwatch.pfeg.noaa.gov.

Heat was more uniformly distributed than chlorophyll levels (Fig. 6). Hence, bleaching was expected to generate uniform large-scale damage patterns with high spatial autocorrelation in coral cover changes among sites within regions. COTS outbreaks were determined by heterogeneously distributed chlorophyll levels and by landscape morphology. Many reefs in the Farasan Banks and Islands are separated by hundreds of meters of water-depth. Combined with patchy chlorophyll highs and settlement stochasticity of COTS, this caused patchy pattern of damage and low spatial autocorellation of coral-cover among sites.

**Figure 7:**
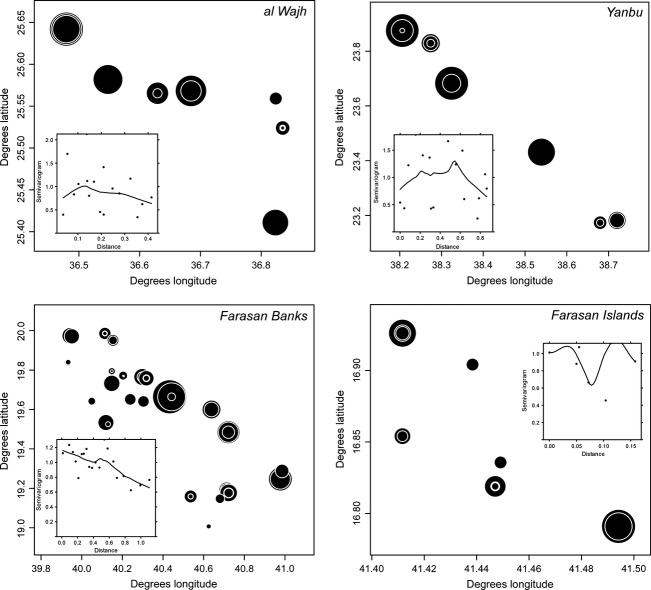
Spatial distribution of coral cover on sampled reefs. The four panels show the geographical spread of sites in the four sampled regions. Size of the bubble is relative to percent coral cover in the site. Several concentric circles show overlapping sites (overlap is due to coarse geographic resolution) with different cover. No systematic differentiation of high- and low-cover sites is observed, suggesting that disturbances leading to low cover act locally. Transects are from equivalent environments and comparable depth. The small inset graphs show the semivariograms of spatial autocorrelation, which show disorganized scatter.

Coral cover ranged from 1% to 98% (Mean 31 ± 19 SD%) on ecologically equivalent reefs. Fig. 7 shows spatial distribution of disturbed and undisturbed sites. Damaged and undamaged reefs were situated in immediate vicinity and no spatial autocorrelation existed in cover values (Fig. 7 insets).

### Will disturbances increase?

COTS outbreaks are triggered by high phytoplankton biomass releasing starfish larvae from food limitation (Birkeland 1982; Houk et al. 2007; Fabricius et al. 2010). Starfish exhibit maximum fertility during the winter-months, and settled larvae switch to corals as diet after 4–6 months (Yamaguchi 1974). About 6 months preceding COTS outbreaks, raised chlorophyll levels were observed at Wajh Banks (Fig. 6). Farasan Banks and Islands are frequently characterized by high chlorophyll concentrations, moving with fronts associated with a cyclonic gyre (Acker et al. 2007). Climate change (Raitsos et al. 2011) may alter its position and dynamics, potentially also modifying COTS dynamics. Rampant coastal development is increasing coastal pollution and nutrient input. It is possible that COTS outbreaks may become more frequent in the future.

**Figure 8:**
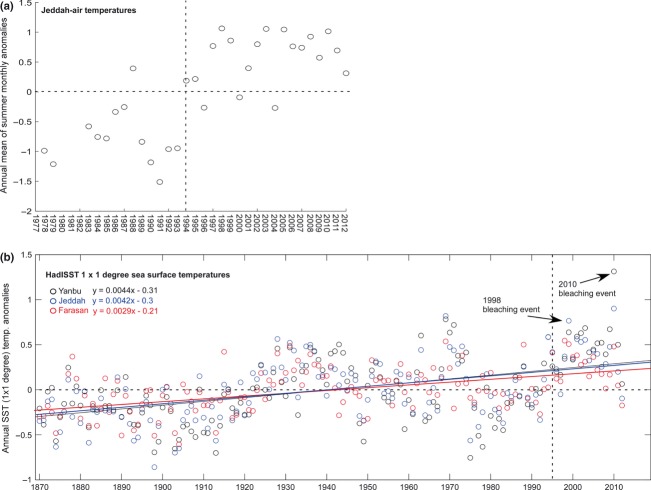
Temperature data showing warming of the Red Sea. (a) Annual means of summer monthly anomalies (July–September, the period in which bleaching is observed) of air temperatures in Jeddah. Since 1994, virtually all annual anomalies are positive. (b) Annual anomalies of sea-surface temperatures (HadISST2 data, data under Crown copyright) across the sampled area. Also here, since 1994 virtually all anomalies are positive, indicating warming of the Red Sea. This pattern is concordant with findings by Raitsos et al. (2011). The warming trend is strongest in the northern Red Sea.

Bleaching events occur during abnormally warm years and heat waves will increase in frequency (Nasrallah et al. 2004). Air temperature data from Jeddah indicated that since 1994 annual summer anomalies were almost exclusively positive (Fig. 8A). Air temperatures are highly correlated with water temperatures on reef flats and in shallow lagoons (*R*^2^ = 0.98). HadISST2 sea-surface temperature showed the same trend (Fig. 8B). From 1994 onward, almost all annual anomalies were positive, while in the period back to 1870, they were mostly negative. Warming trends are strongest in the northern Red Sea at Yanbu and weakest in the already warm southern Red Sea at Farasan Islands. The 2010 bleaching event showed as an unusually strong anomaly, but appeared restricted to the northern Red Sea (Fig. 8B). The observed warming trend agrees with findings by Raitsos et al. (2011) from oceanographic data. Thus, increased future bleaching frequency must be expected.

Widespread reduction of *Acropora*, *Pocillopora,* and other fast-growers at the expense of *Porites*, faviids and other slow-growers (Table 3) suggested potential changes of community structure. Recruitment and impacts suggested that slow-growers might potentially dominate reefs in future. But on inshore reefs in 2010, more slow-growers died than in fast-growers (Table 2). To obtain more clarity about likely population trajectories, longer term consequences of disturbances were simulated in model.

Models assumed that:

- All sites will be disturbed, but intervals between disturbances and severity can vary among sites.
- Recruitment in fast-growers (geometric mean 0.7) is lower than in slow growers (geometric mean 1).
- Disease mortality is low (1.5% in fast-growers vs. 1% of slow-growers).
- Connectivity assumption: focal population exports 50% of recruits and receives 50% of connected population. Latter can be disturbed simultaneously to the same level as focal population, or remain undisturbed (resulting in greater availability of recruits).
- Populations can be connected or isolated.

### Modeling effects of frequent disturbances

**Figure 9:**
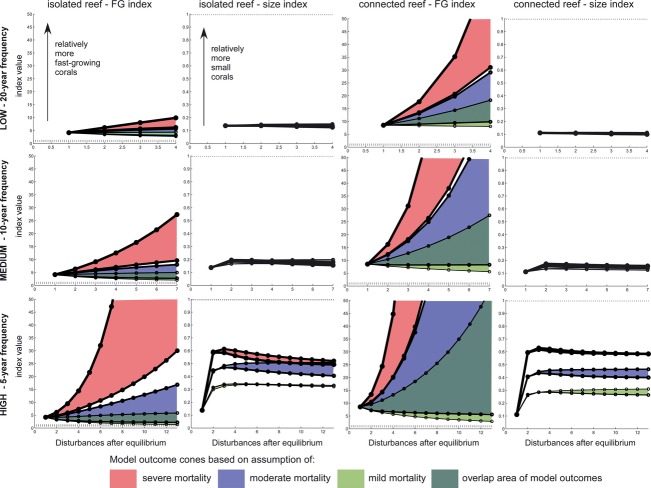
Model sensitivity to disturbance severity and frequency. Outcome cones based on model parameterization shown in Table 1 (upper line representing equal mortality, lower line higher mortality in fast growers). Unless mortality is preferentially in fast-growers, they increasingly dominate reefs. In mild and moderate disturbance scenarios, slow-growers can become dominant. Under severe disturbance frequency and severity, a weedy community of fast-growing small corals is established, but in mild and moderate disturbance and severity, slow-growers persist or even dominate. The y-axes were truncated at FG-index 50 for reasons of graphical clarity and since clear dominance was established.

Models were analysed for expected coral dynamics in response to increasing frequency and severity of disturbance. A habitat favoring fast-growers was assumed, typical of windward *Acopora* and *Pocillopora* dominated Red Sea reefs (the majority of sites, Fig. 3). Median fast-grower dominance of 7:1 (observed range 500:1–2:1) was used in models. Recruitment rates were tuned to field results (Table 1). Mortalities were varied according to three scenarios – mild, medium, severe (Table 1, Fig. 9). Connectivity to an equally disturbed or to an undisturbed population was assumed. Undisturbed model populations were dominated by fast-growers within ∼10 years due to high recruitment and faster extension and reached equilibrium (*dC*_*i*_/*dt* = 0) after ∼50 years. At this point disturbances were introduced. Model mortality at disturbances either favored slow-growers or fast- and slow-growers suffered similar levels. Population trajectories were simplified into the FG-index (1 = equal cover, >1 fast-grower dominance) and the ratio of small to big corals. Models were sensitive to disturbance frequency, relative mortality (weighted toward any coral type or uniform), and connectivity resulting in distinct predicted trajectory cones (Fig. 9).

Event recurrence at 20 years had little effect (outcomes-cone doubling or halving the FG unit, Fig. 9) in the isolated setting. With connectivity, the overall larger pool of fast-growers for recruitment caused their increasing dominance (as is realistic for windward reefs in the Red Sea). The size indices did not change.

Equal mortality in fast- and slow-growers increased FG-index in most scenarios, notwithstanding differences in event timing and connectivity (Fig. 9). If mortality was equal among fast- and slow-growers, the latter could become dominant. But the outcome cones generally suggested that mortality was too severe and too closely spaced to allow regeneration of slow-grower populations when faced with fast-growers' competitive superiority, maintained fertility due to shorter juvenile phase and rapid growth. This indicates that slow-growers may in the long run not withstand equal mortality as fast-growers and will decline dramatically resulting in a community of increasingly “weedy” corals (sensu Darling et al. 2012). This was most dramatic and accentuated in high-mortality/high frequency scenarios and when connectivity to an undamaged reef maintained the fertility of the populations (Fig. 9). The trend toward “weedy” species in higher-disturbance scenarios was also visible by the decline in coral size at 5-year disturbance frequency.

### Argument for/against management intervention

Status-quo can be maintained if disturbances are mild, spaced at least 20 years and recruitment patterns unaltered. Disturbances disadvantaging fast-growers (mild-to-moderate bleaching and predator outbreaks) lower coral cover and shift community composition toward slow-grower dominance. Severe or non-specific impacts that damage all corals equally result in dominance by weedy fast-growers. Such disturbances are COTS infestation, and to a lesser extent bleaching. Since frequency of damage recurrence is critical to outcomes, management interventions like COTS removal as a last resort but preferably reduction of factors that favor the development of COTS outbreaks and also predispose corals to bleaching (nutrification, Wiedenmann et al. 2013) will have a strong impact on overall population trajectories by shifting them into the lower-frequency realm (Fig. 9). With global change set to increase disturbance frequency, management intervention will be required to control this frequency to avoid changes in population trajectories.

## Discussion

This study from the Red Sea, an area with a typically Indo-Pacific coral fauna, growth-rates, community dynamics and similar impacts (Dullo 2005; Pratchett et al. 2009; Cantin et al. 2010; De'ath et al. 2012; Kayal et al. 2012) provides universally applicable and general lessons for the future of coral reefs. Increased severity and frequency of disturbance will lead to a loss of presently established communities and the rise of unique new ones. The climate and environmental stage has already been set for these changes to occur (Fig. 8), at least in the Red Sea. Management intervention to slow-down degradation should be considered.

As in other parts of the world (Wilkinson 2008; De'ath et al. 2012), stress and degradation are easily detectable on Saudi Arabian reefs, even those remote from human usage. A third were degraded by natural causes (COTS in 2006 Farasan Islands, 2008 Wajh Bank, 2009 Farasan Banks; bleaching in 2010 Thuwal/Yanbu), some repeatedly (first bleaching, then COTS; Table 3). Impacts occurred in all habitats and reef zones, but the absence of region-wide events had created a mosaic of disturbed and undisturbed patches among and within sites (Fig. 7). In step with climate (increased heat content as trigger for bleaching events, Fig. 8) and environmental (higher nutrient input due to increased coastal population) changes, modifications in disturbance frequency and severity must be expected. Present community composition can only be maintained at >20-year disturbance frequency and with uneven mortality among coral types, that is fast growers suffer more (Baker et al. 2008; Pratchett et al. 2009; Kayal et al. 2012). Reinicke et al. (2003) calculated turnover rates for slow-grower-dominated communities at Sanganeb (same latitude as Farasan Banks) that support our model results and monitoring in Sudan (Schuhmacher and Mergner 1985; Reinicke et al. 2003) suggests “natural” disturbance frequencies >20 year. Since a 10-year, or even 5-year, disturbance frequency must be considered a plausible near-future scenario (Raitsos et al. 2011), changes in population trajectories as modeled herein might occur on Red Sea reefs over the next few decades. Indeed first signs for such dynamics exist not only in the Red Sea (Riegl et al. 2012), but also elsewhere in the Indo Pacific (De'ath et al. 2012).

Model outcomes reflected the advantages of r-selection and high connectivity in ephemeral habitats (see also Darling et al. 2012), hence small fast-growers increasingly dominate with more frequent and severe disturbance. At higher disturbance frequency (Donner et al. 2009; Raitsos et al. 2011), entire reefs must be considered ephemeral habitats analogous to present reef flats dominated by fast growing *Acropora*, *Pocillopora,* and *Stylophora* - species characterized as weedy by Darling et al. (2012). Habitat preferences in settlement (Suzuki et al. 2008) make it unlikely that entire reefs will be dominated by the same suit of species, thus the fast-growing winners will likely comprise many species. On medium-deep (10–20 m) reefs, small massives (slow-growing *Leptastraea, Goniastraea*, *Porites*) still made up the majority of recruits but covered less space than fast-growers (*Acropora* tables, *Echinopora*, *Montipora*). Slow-growers will be denied dominance by the brevity of the disturbance-free interval, if projected disturbance frequency becomes a reality. In the Farasan Islands, the southernmost and hence warmest region that also had the highest proportion of damaged sites (COTS plus bleaching), most sites at all depths were dominated by fast-growers, thus representing the community shift suggested by the models.

Connectivity is shown as a key factor determining model behavior and regular inter-reef exchanges will be required to maintain unchanged coral communities. In the more moderate scenarios, connectivity to larger (undisturbed) populations changed trajectories markedly from those connected to equally disturbed and hence shrinking populations (Fig. 9). Region-wide disturbance, or indeed any factor interfering with connectivity or depressing the availability of larvae (Munday et al. 2009) would therefore reduce reef resilience. But the intricate pattern of neighboring disturbed and undisturbed reefs (Fig. 7) suggested locally-focused depredation by COTS as main agent of decline, rather than region-wide bleaching. Mortality observed due to COTS (10–99%) exceeded that by the 2010 bleaching event (10–50%), at least on offshore reefs, and infestations were observed in all survey years versus “only” two known bleaching events (1998, 2010). Thus, due to the apparently high frequency of outbreaks and the considerable damage done, COTS appear presently to be the more significant threat to Red Sea coral populations. Our models support De'ath et al. (2012) who show that managing COTS outbreaks could at least arrest declines, if not even increase coral cover on the Great Barrier Reef. Increased heat represents a significant threat that is hard to manage, but COTS outbreaks could potentially be managed in the Red Sea by managing water quality (Fabricius et al. 2010) and conceivably also by removing the starfish. Reducing nutrient inputs could also reduce bleaching sensitivity (Wiedenmann et al. 2013). By thus reducing the severity and frequency of disturbances, population declines and community changes could be arrested or at least slowed down and coral trajectories could conceivably be maintained within the mild disturbance/low frequency realm (Fig. 9). Fewer severe disturbances would also help to maintain connectivity among less damaged sites, thus raising population resilience over all.

If no action is taken, disturbance severity and frequency will increase further, as predicted by global change scenarios. This will push Red Sea reefs further along the trajectory of alteration and degradation of the coral communities. The warning signs appear clearly in field data and models.
